# Effects of Parenteral Vitamin D on the Biomarkers of the Endothelial Function in Patients with Type 2 Diabetes and Ischemic Heart Disease: A Randomized Clinical Trial

**Published:** 2018

**Authors:** Azita H. Talasaz, Elnaz Shaseb, Maryam Tohidi, Farzad Hadaegh, Hamid Ariannejad, Mohammad Abbasinazari

**Affiliations:** a *Department of Clinical Pharmacy, Faculty of Pharmacy, Tehran University of Medical Sciences, Tehran, Iran.*; b *Tehran Heart Center, Tehran University of Medical Sciences, Tehran, Iran.*; c *Department of Clinical Pharmacy, Faculty of Pharmacy, Tabriz University of Medical Sciences, Tabriz, Iran.*; d *Prevention of Metabolic Disorders Research Center, Research Institute for Endocrine Sciences, Shahid Beheshti University of Medical Sciences, Tehran, Iran.*; e *Department of Clinical Pharmacy, School of Pharmacy, Shahid Beheshti University of Medical Sciences, Tehran, Iran.*

**Keywords:** Vitamin D, Ischemic heart disease, Endothelial function, Diabetes mellitus

## Abstract

Vitamin D deficiency is associated with cardiovascular and metabolic diseases. Cardiovascular diseases, in turn, are responsible for mortality of patients with type 2 diabetes (T2D). We investigated whether a single parenteral dose of 25(OH) Vit D could improve the endothelial function in T2D patients with ischemic heart disease. A randomized, placebo-controlled, and double-blind trial was performed on 112 patients randomly divided into vitamin D (n = 55) and placebo (n = 57) groups. A randomization table was used to administer a single dose of either vitamin D (300000 IU) or a matching placebo, intramuscularly. The levels of 25(OH) Vit D, intercellular adhesion molecule 1 (ICAM-1), and vascular cell adhesion molecule 1 (VCAM-1) were measured at baseline and at 8 weeks. In the supplemented group, the level of serum 25(OH) Vit D was increased significantly (29.6 ± 20.8 *vs.* 44.5 ± 19.2 ng/mL; *P* < 0.05), whereas no changes were observed in the placebo group. Within the supplemented group, before and after vitamin D intervention no significant changes in the levels of ICAM-1 and VCAM-1 were observed. The marginal means of the outcome variables (ICAM-1, VCAM-1, and 25(OH) Vit D) were compared between the groups using ANCOVA, adjusted for the baseline of each variable itself: no significant difference was seen in the markers of the endothelial function. A single parenteral dose of vitamin D in T2D patients with ischemic heart disease failed to show improvement in endothelial function.

## Introduction

It is estimated that developing countries in Asia and in the Middle East will have the largest increase in the prevalence of diabetes mellitus by 2030 and the incidence of type 2 has doubled in recent years (1). Cardiovascular diseases are the leading cause of death among patients with type 2 diabetes (T2D) (2). According to experimental studies, deficiency in the serum levels of 25-hydroxy vitamin D [25(OH) Vit D] (<20 ng/mL) (3) is associated with cardiovascular and metabolic diseases such as diabetes mellitus, hypertension, and coronary artery disease (4). Not only does 25(OH) Vit D play a critical role in the musculoskeletal system but also it can exert regulatory effects on the function of the cardiovascular system via vitamin D receptors expressed in nonskeletal tissues such as the heart, kidney, and adipose tissue as well as in lymphocytes (4, 5). One of the mechanisms by which 25(OH) Vit D may protect against cardiovascular disorders is the down regulation of the renin-angiotensin system, conferring protective effects in the form of cardiac remodeling, and blood-pressure regulation (6). Vitamin D may down-regulate the expression of the pro-renin gene, and also may affect other renin-angiotensin-aldosterone system (RAAS) components (7). Furthermore, vitamin D may inhibit the proliferation of cardiomyocytes and vascular smooth muscle cell (8). Given that inflammation contributes to the pathogenesis of cardiovascular diseases, the anti-inflammatory action of vitamin D may explain its positive effects on the cardiac vasculature (8, 9).

The endothelial function as a marker of the vascular system is a good predictor of cardiovascular risk (10). Irregularity in the endothelial function is involved in the pathogenesis of cardiovascular diseases and T2D (11). Hypovitaminosis D is linked to arterial stiffness and impaired endothelial function (12-14). A rise in the serum levels of cellular adhesion molecules, intercellular adhesion molecule 1 (ICAM-1), and vascular cell adhesion molecule 1 (VCAM-1) as specific markers of endothelial dysfunction indicates an impaired endothelial function (11). In observational studies, a direct association has been demonstrated between low serum levels of 25(OH) Vit D and endothelial dysfunction (13). 

The aim of the current study was to determine whether the administration of a single dose of vitamin D could improve the endothelial function as ascertained via the measurement of the levels of adhesion molecules in T2D patients with ischemic heart disease (IHD).

## Experimental

*Patients and design*

The present randomized, placebo-controlled, and double-blind study was conducted on 112 patients aged between 18 and 65 years with known T2D and established IHD. Subjects were recruited from the diabetes clinics of teaching hospitals affiliated with Shahid Beheshti University of Medical Sciences, Tehran, Iran. The study protocol was approved by the Ethic Committee of Shahid Beheshti University of Medical Sciences (8ECRIES-2013), and written consent was obtained from each participant before enrolment. The study was registered at the Australian New Zealand Clinical Trial Registry (ACTRN12614000529640). The patients were excluded if they were on medications and supplements that could inﬂuence vitamin D (glucocorticoids, anti-rejection medications, anticonvulsants, and vitamin D supplements). The other exclusion criteria comprised pregnancy and breastfeeding, history of abnormal liver function tests, renal failure, hypersensitivity to vitamin D or any component of the formulation, hypercalcemia, nephrolithiasis, and rheumatoid diseases. The patients were instructed not to change their lifestyle and the type or dosage of their current medications during the 2-month trial period. 

Through a randomization table, the participants were randomly assigned to receive either vitamin D (300000 IU) or a matching placebo (Darou Pakhsh, Iran) as a single dose, intramuscular injection. Both participants and investigators were blinded to the treatment randomization throughout the study. An online statistical computing web program was utilized to randomize participant placement (www.graphpad.com/quickcalcs/randomize1.cfm). The drug and the placebo were prepared by the pharmacy of the hospital in order to keep the participating clinicians blind to patient allocation. After the selection of eligible patients, the pharmacy delivered the medications labeled with patient codes, depending on their allocation. Preliminary data of this study findings had been published in Acta Diabetologica (15).

**Table 1:** Demographic and clinical characteristics of the study participants

	**Placebo group (n = 57)**	**Vitamin D group (n = 55)**	***P*** **-value**
Age (y)	55.89 ± 5.24	54 ± 6.13	0.081
Gender			
Female	36 (63.2)	38 (69.1)	0.553
Duration of disease (y)	3.6 ± 1.7	3.9 ± 2.2	0.461
Smoking	14 (24.6)	11 (20)	0.363
BMI (kg/m2)	26.96 ± 2.86	27.47 ± 2.79	0.346
HbA1c (mmol/mol)	7.5 ± 1.6 (59.0 ± 18.0)	8.2 ± 2.0 (66.3 ± 21.8)	0.044
FBS (mg/dL)	168.1 ± 67	186.5 ± 64	0.111
25(OH) Vit D (ng/mL)	31.7 ± 18.1	29.6 ± 20.8	0.373
Medications			
Insulin	24 (42)	19 (34.5)	0.442
Oral hypoglycemic agents	57 (100)	55 (100)	1
Antihypertensive agents	57 (100)	55 (100)	1
Statin	57 (100)	53 (96.3)	0.239
ASA	55 (96)	53 (96)	1
Nitrate	46 (80)	45 (81)	1

BMI: Body mass index; HbA1c: Glycosylated haemoglobin; FBS: Fasting blood sugar; 25(OH) Vit D: 25-hydroxy vitamin D; ASA: Acetylsalicylic acid.

* Data are presented as mean ± SD or n (%).

**Figure 1:**
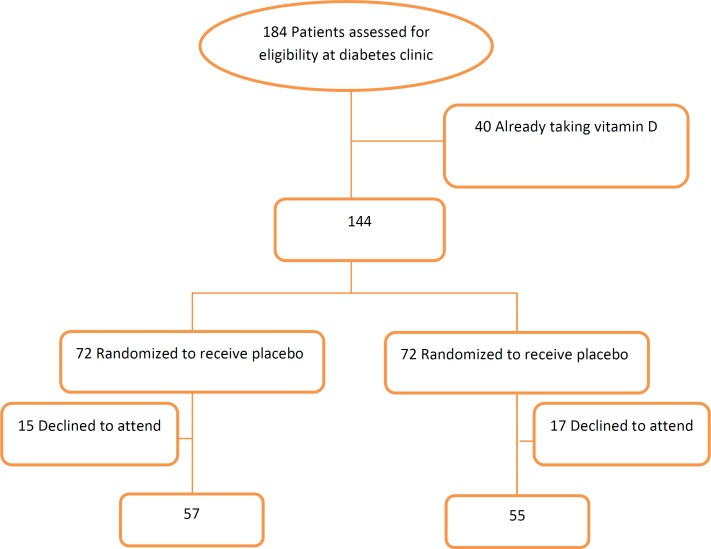
Consort flow chart for study population

*Laboratory Methods*

Fasting blood samples were obtained at baseline (immediately prior to injection) and at 8 weeks and stored at -80 °C for the measurement of the levels of 25(OH) Vit D, ICAM-1, and VCAM-1. The level of 25(OH) Vit D was measured by electrochemiluminescence immunoassay using a Cobas e-411 analyzer and related kits (Roche Diagnostics GmbH, Mannheim, Germany). The intra- and inter-assay coefficient of variation was 2.4% and 6.2%, respectively. The level of VCAM-1 was measured by enzyme immunometric assay using a Sunrise microplate (Tecan Co. Salzburg, Austria) with a kit (Human sVCAM/ CD106, ELISA, Diaclone, France); the coefficient of variation of the intra- and inter-assay was 2% and 2.8%, correspondingly. The level of ICAM-1 was measured by enzyme immunometric assay using a commercial kit (Human CD54/ ICAM-1, ELISA, Diaclone, France) and a Sunrise ELISA reader (Tecan Co. Salzburg, Austria); the coefficient of variation of the intra- and inter-assay was 2.7% and 3.9%, respectively. The quantities were reported in ng/mL for all the above mentioned parameters.

*Statistical Analysis*

The data are presented as means (standard deviations) for the continuous variables and as numbers (%) for the categorical variables. The paired *t*-test was used for within-groups comparisons of the mean of the levels of 25(OH) Vit D, ICAM-1, and VCAM-1. Additionally in the current study, since the results of the measurements of HbA1c and endothelial factors were reported, these variables were compared between the vitamin D and placebo groups using ANCOVA to adjust for the baseline value of each variable itself and HbA1c. (The results are reported as adjusted marginal means). The data analyses were performed using SPSS, version 18 (SPSS Inc, Chicago, IL), and a *P*-value equal to or less than 0.05 was considered statistically significant.

## Results

Out of 184 patients screened for the inclusion in the present study, 144 patients were eligible. The patients were randomized to the vitamin D group or the placebo group. Thirty two out of the 144 patients withdrew from the study. Finally, 57 patients in the placebo group and 55 patients in the vitamin D group completed the study. The Flowchart of the study is depicted in Figure 1, and the baseline characteristics of the participants are presented in Table 1. There were no significant differences in the baseline values among the groups. In the supplemented group, the level of serum 25(OH) Vit D was increased significantly (29.6 ± 20.8 *vs.* 44.5 ± 19.2 ng/mL; *P* < 0.05), while no changes were observed in the placebo group. The comparisons of the measured markers within the groups before and after vitamin D intervention showed no significant changes in the levels of ICAM-1 (624.1 ± 269.8 *vs.* 588.3 ± 247.23 ng/mL) and VCAM-1 (1812.2 ± 641.2 *vs.* 1726.7 ± 637.2 ng/mL) in the supplemented group. In addition, the marginal means of the outcome variables (ICAM-1, VCAM-1, and 25(OH) Vit D) were compared between the intervention and placebo groups using ANCOVA, adjusted for the baseline of each variable itself: no significant differences were detected in the markers of the endothelial function (ICAM-1 = 569.5 [17.63] ng/mL and VCAM-1 = 1745.1 [54.3] ng/mL). No adverse effects were observed in the present study.

## Discussion

What distinguishes the present study from a number of other similar investigations is its target population, drug form selection, and administered dose. To the best of our knowledge, this is the first randomized controlled trial to investigate the effects of plain vitamin D supplementation on the parameters of the endothelial function in T2D patients with established IHD.

Vitamin D deficiency as global health problem was shown to be a modifiable explanatory factor to improve outcomes in several chronic diseases (16). The prevalence of suboptimal vitamin D status among our study subjects was 58%, which is interestingly lower than what was reported (80%) for the Iranian general population in 2012 (17).

The adverse effects of vitamin D deficiency and its association with non-communicable disorders such as diabetes, hypertension, and cardiovascular diseases have already been established. A meta-analysis reported that the incidence of diabetes increased significantly in low levels of vitamin D (18). 

In cross-sectional studies, insulin resistance, hyperglycemia, and T2D have been correlated with low levels of vitamin D (19, 20). A meta-analysis by Parker *et al*. (21) revealed a link between high levels of vitamin D and a 43% decrease in cardiovascular diseases, T2D, and metabolic syndrome. Although according to observational studies vitamin D reduces the risk of diabetes, the results of interventional studies are conflicting; this may be attributed to differences in study populations, study durations, and dosages and drug forms of supplemented vitamin D as well as small sample sizes.

In the current study, we observed that the administration of a single dose of 300000 IU of parenteral vitamin D failed to induce significant changes in the ICAM and VCAM endothelial cell surface markers.

Highly prevalent in Iran and across the globe (22, 23), T2D contributes to damage to both small vessels and larger vessels and ultimately begets microangiopathy and macroangiopathy. Hyperinsulinemia and oxidative stress are both factors implicated in the pathogenesis of diabetes-related vascular complications (24). Endothelial dysfunction is the underlying cause of diabetic angiopathy, which eventually leads to cardiovascular diseases (25). According to a study, the biomarkers of endothelial dysfunction can predict the incidence of T2D independent of known risk factors for diabetes (26).

Clinical studies have indicated that vitamin D deficiency is associated with an increased risk of cardiovascular diseases (27-29). In view of the fact that endothelial dysfunction is known to promote cardiovascular diseases, vitamin D can be effective in the improvement of the endothelial function. Several observational studies have elucidated the link between vitamin D deficiency and vascular diseases, documenting an increased risk of cardiovascular events with lower levels of vitamin D, while only a few have assessed the impact of low vitamin D levels on the endothelial function.

In a similar randomized clinical trial to ours, Sugden, *et al.* (30) showed that a single dose of 100000 IU of oral vitamin D improved the endothelial function in their patients with T2D and vitamin D insufficiency which is in disagreement with our findings. Even though the two studies are similar in some aspects, they differ with respect to some other properties, all of which might account for the different results obtained; first the study population was different. The subjects in our study were Iranian T2D patients with established IHD whereas their subjects were Scottish T2D patients, not particularly with IHD. Second, the drug form, dosage and route of administration differed in the 2 studies (300000 IU parenteral vitamin D3 *vs.* 100000 IU oral D2 (Ergocalciferol)). Third, we didn’t limit the patient enrollment to any specific season while they enrolled all the patients in winter. In order to better adherence, we prefer to administer parenteral form of vitamin D in this study. Nitin Gupta *et al.* have reported that both oral (60000IU/d) and IM (300000 IU) are effective for the treatment of Vitamin D deficiency. They have reported that vitamin D levels in the IM group showed a sustained increase from baseline too (31). Another study by Tacrin *et al.* (32) also supported the idea that vitamin D replacement in individuals with 25(OH) Vit D deficiency had a favorable effect on their endothelial function. Elsewhere, Shab-bidar *et al.* (25) demonstrated that the biomarkers of the endothelial function were improved following regular vitamin D intakes in their subjects with T2D. In contrast to our results, the 3 aforementioned studies showed a positive relation between vitamin D replacement and the endothelial function. Nonetheless, our results chime in with those reported by the following 2 investigations. In a randomized clinical trial focusing on postmenopausal women, Wood *et al.* (33) sought to determine whether daily doses of vitamin D at 400 or 1000 IU/d for 1 year could affect the conventional markers of cardiovascular disease risk and reported that sICAM, a biomarker of the endothelial function, was not affected by vitamin D treatment. In a trial performed by Witham *et al. *(34), vitamin D supplementation in both 100000 and 200000 IU IM did not significantly influence the endothelial function as assessed by flow-mediated dilatation. So in present study, we decided to administer higher dose (300000IU) than doses of Witham et al study in order to get a better response. 

*Strengths and Limitations*

Salient among the strong points of the present study are its double-blind, randomized, clinical design, and acceptable level of subject compliance. Nevertheless, the limitations of our study should be taken into account in the interpretation of its results. First and foremost, that the duration of our study was 8 weeks and our study sample was comprised of a small number of participants means that its results cannot be generalizable to longer periods and larger patient populations. It is also worthy of note that the findings of our study can only be applied to T2D patients with IHD and may not be applicable to those without IHD.

## Conclusion

In the present study, a single intramuscular injection of 300000 IU of vitamin D in T2D patients with IHD failed to induce changes in the biomarkers of the endothelial function.
